# Clinical implications of malnutrition in Huntington's disease progression: evidence from a Chinese cohort and Mendelian randomization

**DOI:** 10.3389/fnut.2026.1718264

**Published:** 2026-03-23

**Authors:** Jie-Qiang Xia, Yang-Fan Cheng, Si-Rui Zhang, Yuan-Zheng Ma, Jia-Jia Fu, Tian-Mi Yang, Ling-Yu Zhang, Jean-Marc Burgunder, Hui-Fang Shang

**Affiliations:** 1 Department of Neurology, Laboratory of Neurodegenerative Disorders, Rare Disease Center, West China Hospital, Sichuan University, Chengdu, China; 2 Department of Neurology, The First People's Hospital of Shuangliu District, Chengdu, China; 3 Department of Neurology, Swiss Huntington's Disease Center, Siloah, University of Bern, Bern, Switzerland

**Keywords:** CONUT, GNRI, Huntington's disease, malnutrition, nutritional status, PNI

## Abstract

**Background:**

Huntington's disease (HD) is a neurodegenerative disorder associated with progressive motor, cognitive, and psychiatric dysfunction. Peripheral metabolic disturbances, including malnutrition, are commonly observed in HD. However, the prevalence, clinical correlations, and prognostic value of malnutrition in HD, especially among Chinese patients, remain inadequately explored.

**Methods:**

This cohort study recruited 113 genetically confirmed HD patients and 113 age/sex-matched healthy controls (HCs). Nutritional status was assessed using the Controlling Nutritional Status (CONUT) score, Geriatric Nutritional Risk Index (GNRI), and Prognostic Nutritional Index (PNI). Clinical evaluations included Unified Huntington's Disease Rating Scale (UHDRS), cognitive tests, and psychiatric assessments. Kaplan-Meier survival analysis and Cox regression models were used to evaluate the prognostic significance of malnutrition. Mendelian randomization (MR) analysis was employed to explore causal relationships between nutritional indicators and HD progression using genome-wide association study (GWAS) data.

**Results:**

During a mean follow-up of 5.74 years, 44 patients reached composite endpoints of death or loss of independent function [total functional capacity (TFC) ≤ 2]. HD patients showed higher malnutrition prevalence than HCs (CONUT: 34.51 vs. 13.27%; GNRI: 7.96 vs. 2.65%). Malnutrition correlated with functional decline, cognitive impairment, advanced disease stage, and lower composite Unified Huntington's Disease Rating Scale (cUHDRS) score, but not with survival outcomes. MR analysis suggests a causal relationship between lymphocyte count and delayed motor progression in HD patients.

**Conclusion:**

Malnutrition was highly prevalent in Chinese HD patients and was associated with functional and cognitive decline. Although malnutrition did not independently predict survival, MR analysis suggests that lymphocytes delay motor progression, implying that immunonutritional pathways may warrant further investigation as potential targets for future mechanistic and interventional research.

## Introduction

Huntington's disease (HD) is an inherited neurodegenerative disorder characterized by motor impairment, behavioral disturbances, cognitive deficits, and neuropsychiatric manifestations (1). HD typically manifests in middle adulthood and progresses over 15 to 20 years (2). Currently, there is no therapy to modify the disease, resulting in a significant economic strain on both families and society, which intensifies over the progression of the illness (3).

Aside from the well-known central nervous system degeneration, HD exerts broad systemic effects, disrupting peripheral metabolic homeostasis and body composition (4–6). Among these systemic features, unintended weight loss is an early finding in HD (4). Intriguingly, people with HD typically experience CAG-repeat-dependent weight loss (7), a reduced body mass index (BMI), and lower lean body mass (8), despite increased appetite and calorie intake (7). This is more likely caused by increased resting energy expenditure in HD (8). Malnutrition in HD may result from a combination of factors, such as metabolic disturbances, dysphagia, and increased energy expenditure due to involuntary movements (5, 9). In an observational study, body weight has been identified as a predictor of the rate of HD progression, with a higher BMI being associated with slower functional decline, suggesting a protective role of adequate nutrition (10). Malnutrition often leads to a poorer quality of life, increased morbidity, and higher mortality (11, 12). Given the unique metabolic and pathological alterations in HD, evaluating nutritional status is paramount to understanding its relationship with clinical outcomes.

The assessment of nutritional status is inherently challenging due to the complex, multifaceted nature of malnutrition (13). Although guidelines recommend regular nutritional screening, no validated tool exists specifically for HD (9). Several innovative and straightforward nutritional assessment tools, such as the Geriatric Nutritional Risk Index (GNRI) (14), the Prognostic Nutritional Index (PNI) (15), and the Controlling Nutritional Status score (CONUT) (16), have been used to identify malnutrition or the risk of malnutrition and predict outcomes in Parkinson's disease (PD) (17–20), and Multiple system atrophy (MSA) (21), but their applicability to HD is untested. These nutritional assessment tools have also shown prognostic relevance in non-neurological chronic diseases, such as heart failure and chronic kidney disease (22–24). A systematic evaluation of malnutrition and its relationship with clinical outcomes in Chinese patients with HD is lacking. Moreover, the causal relationships between nutritional status and disease progression in HD remain unclear.

This study aimed to determine the prevalence of malnutrition using the GNRI, CONUT, and PNI in a Chinese HD cohort, and to evaluate the associations between malnutrition and clinical features, as well as survival outcomes. Finally, we employ Mendelian randomization (MR) to investigate the causal relationship between nutritional indicators, including albumin levels, BMI, and lymphocyte counts, and HD progression. Our research aims to inform the development of future interventions that target nutritional support, potentially modifying disease progression and improving patient outcomes in HD.

## Methods

### Cohort study

We followed the Strengthening the Reporting of Observational Studies in Epidemiology–Nutritional Epidemiology (STROBE-nut) guidelines for the reporting of cohort studies (25).

### Study population

The study was conducted at the Department of Neurology at West China Hospital of Sichuan University. Hundred and thirteen manifested HD and 113 age-, sex-matched healthy controls (HCs) were recruited for the study. The HCs were matched with the patients in terms of age, sex, and BMI. The participants were part of the same cohort as in a previous study, and all had received approval from the Institutional Ethics Committee of the hospital (2, 26, 27).

The inclusion criteria for HD patients were as follows: CAG repeat length >39, confirmed by a gene test, and motor disturbances, as indicated by a Unified Huntington's Disease Rating Scale (UHDRS) total motor score (TMS) diagnostic confidence score of 4 (28). Patients and HCs were excluded if they had malignant tumors, digestive diseases, autoimmune diseases, hepatic or renal failure, acute or chronic inflammatory diseases, infections at baseline assessment, or other chronic diseases that may have influenced their nutritional status. Additionally, participants were excluded if they were taking medications for lipid-lowering, lacked essential clinical and laboratory data, could not complete the rating scales, or had a positive family history of neurodegenerative diseases (applied to HCs only).

### Demographic and clinical data collection

All participants in the study underwent face-to-face interviews with experienced neurologists. The interviews aimed to collect a range of demographic characteristics, including age, sex, height, weight, educational attainment, family history, smoking habits, and drinking habits. Additionally, the baseline assessment documented the age at which symptoms first manifested, the disease duration, the number of CAG repeats, the presence of comorbidities, and the treatments received.

BMI was calculated as body weight (kg) divided by height squared (m^2^). The composite Unified Huntingtons Disease Rating Scale (cUHDRS) is a valuable tool for evaluating clinical progression, with a higher score indicating a less severe condition (29). Motor function was evaluated using the UHDRS-TMS, while daily functional performance was assessed with the UHDRS-total functional capacity (TFC) scale. A battery of cognitive tests was employed to assess the participants' cognitive function, including the Mini-Mental State Examination (MMSE), Symbol Digit Modality Test (SDMT), Stroop Word Reading Test (SWR), Stroop Color Naming Test (SCN), Stroop Interference Test (SI), and Trail Making Test (TMT) A and B. Additionally, researchers assessed psychiatric symptoms using the Hamilton Depression Scale (HAMD), the Hamilton Anxiety Scale (HAMA), the Beck Depression Inventory (BDI), and the short version of the Problem-Behavior Assessment (PBA-s) (27).

### Malnutrition assessment

The nutritional status of the participants was evaluated using GNRI, PNI, and CONUT scores, which were calculated based on the data collected during the baseline assessment. The initial assessment included blood sampling following an overnight fast. The Department of Laboratory Medicine at West China Hospital, Sichuan University, conducted tests for serum albumin level, total cholesterol, and overall lymphocyte count. Albumin values measured in g/L were converted to g/dl by dividing by 10; lymphocyte counts measured in 10^9^/L were converted to cells/mm^3^ by multiplying by 10^3^ prior to PNI calculation. The GNRI is calculated using the following formula: 1.489 ^*^ serum albumin (g/L) + 41.7 ^*^ body weight (kg)/ideal body weight (kg) (14). Ideal body weight was calculated using the formula: 22 ^*^ square of height (m^2^) (17, 21). If the actual body weight is greater than the ideal body weight, the ratio of the body weight to the ideal body weight is set to 1. Based on the GNRI values, four grades of nutrition-related risk have been established: high risk (GNRI < 82), moderate risk (GNRI 82 to < 92), low risk (GNRI 92 to ≤ 98), and no risk (GNRI >98) (14). PNI is calculated using the formula: 10 × serum albumin (g/dl) + 0.005 × total lymphocyte count (mm^3^) (15, 30). The patients were divided into three groups: those without nutritional risk (PNI >38), those with moderate nutritional risk (PNI ranging from 35 to 38), and those who had severe nutritional risk (PNI < 35). There is no mild category for the PNI. The CONUT score is calculated by adding the scores for serum albumin, total cholesterol, and total lymphocyte count (16). The score for serum albumin is based on the following ranges: 0 for ≥3.5 g/dl, 2 for 3.0–3.49 g/dl, 4 for 2.50–2.99 g/dl, and 6 for < 2.50g/dl. The score for total cholesterol is determined as follows: 0 for ≥180 mg/dl, 1 for 140–179 mg/dl, 2 for 100–139mg/dl, and 3 for < 100mg/dL. Lastly, the score for total lymphocyte count is assigned as follows: 0 for ≥1.61 × 10^9^/L, 1 for 1.20–1.591 × 10^9^/L, 2 for 0.80–1.191 × 10^9^/L, and 3 for < 0.81 × 10^9^/L. Patients were grouped according to malnutrition risk: normal (CONUT 0–1), mild (CONUT 2–4), moderate (CONUT 5–8), and severe (CONUT 9–12) (16).

### Endpoints and follow-up

The primary endpoint was a composite of (i) all-cause death or (ii) loss of independent function (defined as TFC ≤ 2) (27), and time-to-event was defined as the baseline assessment date to the first occurrence of either component of the endpoint event. Kaplan–Meier curves and Cox models use the composite endpoint. Patients were regularly followed up by telephone or outpatient visits every 6–12 months to collect information on clinical outcomes. Endpoint information was obtained from scheduled clinical follow-up assessments and, when needed, from family reports and/or medical records.

### Statistical analysis

Statistical significance was set at *p* < 0.05. IBM SPSS software (version 20.0) was used for the statistical analysis. All continuous data are presented as the mean ± standard deviation or median (IQR) as appropriate. All categorical variables are presented as numbers or percentages. For each continuous variable, we used the Kolmogorov–Smirnov test to evaluate the normality of the distribution. Student's *t*-test or the Mann–Whitney *U*-test was used to compare continuous variables between different groups. The chi-square test was performed to compare categorical variables. The multivariate linear regression model was conducted to investigate clinical features associated with nutritional status. Patients with HD were divided into normal nutrition and malnutrition groups based on GNRI (>98 vs. ≤ 98) and CONUT (< 2 vs. ≥2), respectively. In models with malnutrition status (GNRI- or CONUT-defined) as the dependent variable, the independent covariates included disease stage, UHDRS-TMS and TFC, SDMT and cUHDRS. The Kaplan–Meier analysis was used to assess the prognostic significance of the GNRI, PNI, and CONUT scores in relation to survival. Univariate and multivariate survival analyses were performed using the Cox proportional-hazards regression model. Cox proportional-hazards regression was used to evaluate the association between malnutrition status and time-to-event outcomes in HD. We fitted two pre-specified multivariable models, separately including malnutrition defined by CONUT or GNRI. Both models were adjusted for the same covariates: age, sex, motor symptom of onset, disease duration, CAG repeat length, and baseline score of UHDRS-TMS. These covariates were selected *a priori* because they are established or plausible prognostic factors in HD and potential confounders of the malnutrition–outcome association.

### Genome-wide association study (GWAS) summary statistics

All data used in the MR analyses were derived from studies that had already obtained related ethical review board approval and informed consent. The data sources used in this study are detailed in Supplementary Table 1. We conducted a Mendelian randomization (MR) analysis of nutrition-associated indicators with HD based on the formulas for CONUT, GNRI, and PNI to explore the potential causal association between nutrition-associated indicators and the clinical characteristics of HD patients based on previously reported GWAS summary statistics. The genetic instrument associated with albumin was obtained from the UK Biobank (ukb-d-30600) (http://www.nealelab.is/uk-biobank/), which included 432,048 participants, and cholesterol (ukb-d-30690), which included 470,314 participants The genetic instrument associated with BMI was obtained from the GIANT-consortium GWAS study by Yengo et al. in 2018 (31), which identified 510 conditionally independent SNPs. Lymphocyte was obtained from the Blood Cell Consortium by Vuckovic et al. (32). Single nucleotide polymorphisms (SNPs) with minor allele frequency >0.01, reaching genome-wide significance (*p* < 5 ^*^ 10–8) were further clumped to exclude SNPs in linkage disequilibrium based on 1,000 Genomes Project Phase 3 (clumping window of 10,000 kb, r2 < 0.001) (33). In addition, we evaluated the power of SNPs by calculating F statistics for each SNP and excluded SNPs with *F* statistics < 10. The summary-level data for the outcomes of HD were derived from a GWAS meta-analysis conducted by the Genetic Modifiers of Huntington's Disease (GeM-HD) Consortium, which included 9,064 HD patients of European descent (34). Residual age of onset (RAOO) was defined as the difference between the actual AOO (diagnostic motor signs) and expected AOO (calculated based on CAG repeat number). The GWAS additionally utilized predictive modeling to estimate the age at which each individual demonstrated a 50% probability of the pre-determined clinical landmark phenotype, as detailed in the primary report (35). TMS30 was estimated ages in early manifest HD at which each individual progressed up to TMS = 30. We selected RAOO (*n* = 9,009), predicted age at TFC6 (TFC = 6, *n* = 6,900), and predicted age at TMS30 (TMS =30, *n* = 6,897) as outcomes to verify our findings from the observational analysis.

### MR analysis

The principal analyses used the random-effects inverse-variance weighted (IVW) approach (36). We further performed sensitivity analyses using the method of weighted median, weighted mode, and maximum likelihood. The heterogeneity was quantified by Cochran's Q statistics using the method of IVW and MR-Egger, with a *p* value < 0.05 suggesting the presence of significant heterogeneity. The Egger-intercept method was utilized to assess the directional pleiotropy.

## Results

### Baseline clinical characteristics

**Figure 1:**
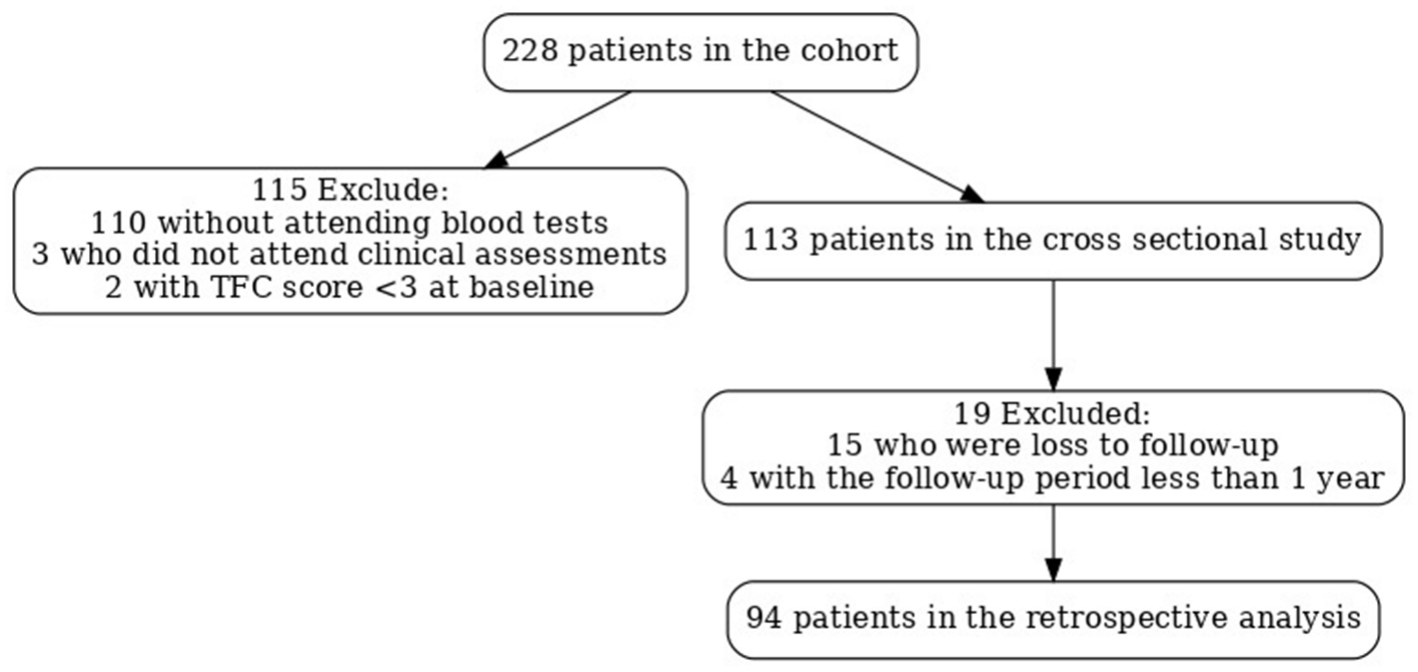
Flowchart of the patient screening process in the study cohort.

After screening 228 HD patients, 113 were included in the cross-sectional study (Figure 1). The patient screening process is shown in Figure 1. Of the 228 patients diagnosed with HD based on genetic testing, 113 were ultimately included in the study. The demographic and hematological data for patients with HD and HCs are presented in Table 1. Age, sex, and BMI were not significantly different between patients with HD and HCs. According to the CONUT score and GNRI, the prevalence of malnutrition in HD was higher when compared to HCs (34.51 vs. 13.27% and 7.96 vs. 2.65%, respectively).

**Table 1:** Demographic and hematological data of patients with Huntington's disease (HD) and healthy controls (HCs) (mean ± SD).

**Variables**	**HD patients**	**HCs**	***p-*value**
Number	113	113	/
Age (*y*)	54.27 ± 11.36	54.05 ± 5.77	0.855
Sex (male, %)	44 (38.9%)	44 (38.9%)	>0.99
BMI	21.29 ± 2.51	21.77 ± 1.42	0.095
Albumin (g/L)	42.99 ± 4.00	46.49 ± 3.12	** < 0.0001** ^*^
Total cholesterol (mmol/L)	4.53 ± 0.91	5.39 ± 0.96	** < 0.0001** ^*^
Lymphocytes (10^9^/L)	1.70 ± 0.52	1.94 ± 0.63	**0.003** ^*^
CONUT score	1.49 ± 1.36	0.62 ± 0.98	** < 0.0001** ^*^
Malnutrition (%)	39 (34.51%)	15 (13.27%)	**0.0003** ^*^
GNRI	103.60 ± 5.88	109.70 ± 5.29	** < 0.0001** ^*^
Malnutrition (%)	9 (7.96%)	3 (2.65%)	0.135
PNI	51.37 ± 5.37	56.19 ± 4.67	** < 0.0001** ^*^

BMI, body mass index; CONUT, controlling nutritional status; GNRI, geriatric nutritional risk index; PNI, prognostic nutritional index; HCs, healthy controls; SD, standard deviation.

^*^Significant difference. The bold values indicate statistically significant results (*p* < 0.05).

### Comparison of demographic and clinical features between HD with normal nutrition and malnutrition

The demographic and clinical features of patients with HD in diverse nutritional statuses according to the CONUT score and GNRI are shown in Table 2. The mean age and age at onset of patients with HD were 54.27 ± 11.36 and 42.73 ± 10.02 years, respectively. Among them, 38.9% of the patients were male. The mean disease duration was 5.41 ± 3.93 years at baseline. Based on the CONUT score, the normal-nutrition group had higher levels of albumin (43.95 ± 3.17 g/L), total cholesterol (4.94 ± 0.83 mmol/L), and lymphocytes (2.01 ± 0.46 × 10^9^/L) compared to the malnutrition group (all *p* < 0.05). There were no significant differences between the two groups in terms of age, sex, BMI, disease duration, age of onset, UHDRS-TMS/TFC score, cUHDRS score, history of smoking and drinking, MMSE score, SDMT, SCN, SWR, SI, PBA-s, HAMD, HAMA, and BDI (all *p* > 0.05). According to the GNRI, compared to the normal-nutrition group, the malnutrition group had lower levels of albumin (37.12 ± 3.57 g/L), UHDRS-TFC scores (5.00 ± 2.83), and cUHDRS scores (3.52 ± 3.19) (all *p* < 0.05).

**Table 2:** Comparison of demographic and clinical features between Huntington's disease (HD) with normal-nutrition and malnutrition.

**Variables**	**CONUT** ^**a**^				**GNRI** ^**b**^		
	**HD**	**Normal nutrition**	**Malnutrition**	***p*** **value**	**Normal nutrition**	**Malnutrition**	***p*** **-value**
Number	113	49	39	/	57	9	/
Age (y)	54.27 ± 11.36	54.93 ± 12.09	53.21 ± 11.23	0.495	53.67 ± 10.75	51.47 ± 11.03	0.572
Sex (male, %)	44 (38.9%)	20 (40.8%)	13 (33.3%)	0.175	18/57 (31.5%)	3/9 (33.3%)	0.963
BMI	21.29 ± 2.51	21.14 ± 2.47	21.78 ± 2.57	0.242	21.11 ± 2.48	21.02 ± 3.30	0.925
CAG repeat	44 (42–46)	44 (42–46)	44 (42–46)	0.509	44 (42–46)	44 (42–46)	0.164
Age of onset (years)	42.73 ± 10.02	42.88 ± 10.59	42.44 ± 9.57	0.839	44.18 ± 8.84	40.29 ± 9.98	0.234
**Family history**							
Maternal inheritance	53/113 (46.9%)	21/49 (42.8%)	18/39 (46.1%)	0.400	22/57 (38.5%)	4/9 (44.4%)	0.769
Paternal inheritance	34/113 (30%)	15/49 (30.6%)	12/39 (30.7%)	0.974	20/57 (35%)	3/9 (33.3%)	0.560
Unclear	26/113 (23%)	13/49 (26.5%)	9/39 (23%)	0.328	15/57 (26.3%)	2/9 (22.2%)	0.789
Genetic anticipation (%)	44.14% (49/111)	37.5% (18/48)	41% (16/39)	0.376	35% (20/57)	33.3% (3/9)	0.789
UHDRS-TMS	38.18 ± 15.76	37.09 ± 15.35	38.26 ± 15.52	0.727	36.89 ± 14.53	42.33 ± 13.83	0.299
UHDRS-TFC	8.13 ± 3.31	8.02 ± 3.10	7.82 ± 3.42	0.777	8.29 ± 3.02	5.00 ± 2.83	**0.003** ^**^
cUHDRS	6.07 ± 3.82	5.97 ± 3.94	5.73 ± 3.73	0.802	6.75 ± 3.17	3.52 ± 3.19	**0.038** ^*^
MMSE	21.61 ± 5.81	21.84 ± 5.51	21.39 ± 5.54	0.734	21.74 ± 5.79	20.00 ± 5.61	0.437
SDMT	15.24 ± 11.26	16.74 ± 11.59	13.13 ± 9.59	0.174	16.18 ± 10.36	10.20 ± 8.56	0.224
SCN	29.41 ± 15.12	28.81 ± 16.45	29.15 ± 13.82	0.926	32.38 ± 14.77	27.00 ± 5.93	0.386
SWR	38.33 ± 19.55	37.49 ± 20.96	37.97 ± 18.08	0.919	42.63 ± 18.18	39.17 ± 14.08	0.658
SI	17.42 ± 11.38	17.81 ± 12.00	15.39 ± 11.58	0.395	17.61 ± 11.32	15.33 ± 12.99	0.653
PBA-s	13.61 ± 13.47	14.26 ± 14.10	13.28 ± 12.04	0.744	14.11 ± 13.96	16.33 ± 14.46	0.661
HAMD	11.17 ± 6.98	12.08 ± 8.33	10.53 ± 4.67	0.470	10.68 ± 7.42	13.86 ± 3.93	0.278
HAMA	8.25 ± 4.16	8.80 ± 4.95	7.33 ± 2.81	0.513	7.92 ± 4.57	9.67 ± 0.58	0.206
BDI	6.87 ± 5.89	8.47 ± 7.46	6.71 ± 4.05	0.426	7.19 ± 6.18	13.50 ± 6.36	0.172
Albumin (g/L)	42.99 ± 4.00	43.95 ± 3.17	41.48 ± 4.64	**0.004** ^**^	44.46 ± 2.77	37.12 ± 3.57	** < 0.0001** ^***^
Total cholesterol (mmol/L)	4.53 ± 0.91	4.94 ± 0.83	3.98 ± 0.75	** < 0.0001** ^***^	4.63 ± 0.90	4.12 ± 0.91	0.122
Lymphocytes (10^9^/L)	1.70 ± 0.52	2.01 ± 0.46	1.34 ± 0.32	** < 0.0001** ^***^	1.70 ± 0.53	1.46 ± 0.47	0.206
PNI	51.41 ± 5.38	54.00 ± 4.37	48.16 ± 4.76	** < 0.0001** ^***^	52.86 ± 4.10	44.42 ± 4.85	** < 0.0001** ^***^
CONUT	1.49 ± 1.36	0.51 ± 0.51	2.72 ± 1.05	** < 0.0001** ^***^	1.41 ± 1.06	2.44 ± 2.13	**0.028** ^*^
GNRI	103.70 ± 5.96	104.80 ± 5.04	102.50 ± 6.67	0.127	105.30 ± 4.26	93.28 ± 3.81	** < 0.0001** ^***^
Smoking (%)	10/47 (21.28%)	4/24 (16.67%)	5/19 (26.32%)	0.226	8/36 (22.2%)	1/7 (14.29%)	0.413
Drinking (%)	11/47 (23.40%)	7/24 (29.17%)	4/19 (21.05%)	0.412	9/36 (25%)	2/7 (28.57%)	0.739
Survival time (years)	9.97 ± 5.18	9.80 ± 5.61	9.63 ± 4.75	0.877	9.76 ± 5.16	11.52 ± 5.45	0.349
Outcome (%)	44/113 (38.94%)	17/49 (34.69%)	18/31 (58.06%)	**0.001** ^**^	24/57 (42.1%)	5/9 (55.55%)	0.09
Final MMSE	12.68 ± 10.58	11.78 ± 10.76	13.31 ± 10.35	0.675	16.65 ± 8.74	21.67 ± 5.77	0.348
ATFC	0.83 ± 1.62	0.57 ± 1.31	1.10 ± 2.00	0.215	0.75 ± 1.42	−0.1 ± 1.79	0.134

CONUT, controlling nutritional status; GNRI, geriatric nutritional risk index; PNI, prognostic nutritional index; BMI, body mass index; cUHDRS, composite unified Huntington's disease rating scale; UHDRS, unified Huntington's disease rating scale; TFC, total functional capacity; TMS, total motor score; MMSE, mini–mental state examination; SDMT, symbol digit modality test; SWR, Stroop word reading test; SCN, Stroop color naming test; SI, Stroop interference test; PBA-s, the short version of the problem-behavior assessment; BDI, beck depression inventory; HAMD, Hamilton depression scale; HAMA, Hamilton anxiety scale.

^*^p < 0.05, ^**^p < 0.01, ^***^p < 0.001.

^*a*^25 were missing data.

^*b*^47 were missing data. The bold values indicate statistically significant results (*p* < 0.05).

### Associations between malnutrition and HD clinical characteristics

The univariate regression models revealed that the GNRI score was negatively correlated with UHDRS-TMS (*r* = −0.253, *p* = 0.044) and disease stage (*r* = −0.319, *p* = 0.009), and positively correlated with UHDRS-TFC (*r* = 0.305, *p* = 0.014) and cUHDRS (*r* = 0.321, *p* = 0.034). The PNI score is positively correlated with SDMT (*r* = 0.286, *p* = 0.019). No significant association was found among the groups based on the CONUT score in the clinical features. The results are presented in Supplementary Table 2. In the multivariate linear regression model, adjust for age, sex, and CAG repeat numbers using different nutritional tools. In the GNRI model, the occurrence of malnutrition in HD patients was associated with a lower TFC score (β = 0.47, *p* = 0.050), a later disease stage (β = −2.38, *p* = 0.012), a lower SDMT score (β = 0.17, *p* = 0.039), and a lower cUHDRS score (β = 0.61, *p* = 0.011). In the PNI model, lower cUHDRS scores (β = 0.33, *p* = 0.047) were associated with malnutrition in patients with HD. Details of related factors are demonstrated in Table 3.

**Table 3:** The associations between malnutrition and Huntington's disease (HD) clinical characteristics by multivariate analysis after adjusting for sex, age, and CAG repeat numbers.

**Clinical characteristic**	**CONUT score**			**GNRI score**			**PNI score**		
	β	**95% CI**	***p*** **-value**	β	**95%CI**	***p*** **-value**	β	**95% CI**	***p*** **-value**
UHDRS-TMS	0.01	−0.01 to 0.03	0.438	−0.10	−0.20 to 0.00	0.057	−0.03	−0.11 to 0.04	0.389
UHDRS-TFC	−0.01	0.10 to 0.09	0.924	0.47	0.00 to 0.93	**0.050** ^*^	0.06	−0.30 to 0.43	0.733
Disease stage	0.15	−0.25 to 0.55	0.459	−2.38	−4.22 to −0.53	**0.012** ^*^	−0.83	−2.31 to 0.66	0.272
SDMT	−0.02	−0.05 to 0.01	0.196	0.17	0.01 to 0.32	**0.039** ^*^	0.11	−0.01 to 0.23	0.060
cUHDRS	−0.05	−0.13 to 0.04	0.314	0.61	0.15 to 1.07	**0.011** ^*^	0.33	0.04 to 0.65	**0.047** ^*^

CONUT, controlling nutritional status; GNRI, geriatric nutritional risk index; PNI, prognostic nutritional index; CI, confidence interval; cUHDRS, composite unified Huntington's disease rating scale; SDMT, symbol digit modality test; TFC, total functional capacity; TMS, total motor score; UHDRS, unified Huntington's disease rating scale.

^*^p < 0.05. The bold values indicate statistically significant results (*p* < 0.05).

### Survival analysis of CONUT score and GNRI in patients with HD

Kaplan–Meier survival analysis showed no significant differences in time to the composite endpoint (death or TFC ≤ 2) across nutritional status categories defined by CONUT or GNRI (Supplementary Figure 1). The mean follow-up time was 5.74 ± 4.52 years. Finally, 44 participants developed either death or a TFC score of 0 to 2. The median survival time was 13.87 years. We performed the univariate and multivariable Cox proportional-hazards regression analysis to demonstrate the predictive value of malnutrition on survival in HD (Supplementary Table 3). In multivariable Cox models adjusted for age, sex, symptom of onset, disease duration, CAG repeat length, and baseline UHDRS-TMS score, malnutrition defined by CONUT was not significantly associated with survival (HR = 1.362, 95% CI 0.505–3.675, *p* = 0.542). Similar results were observed for malnutrition defined by GNRI (HR = 0.418, 95% CI 0.081–2.142, *p* = 0.295).

### The causal association between nutrition-associated indicators and disease clinical characteristics

**Figure 2:**
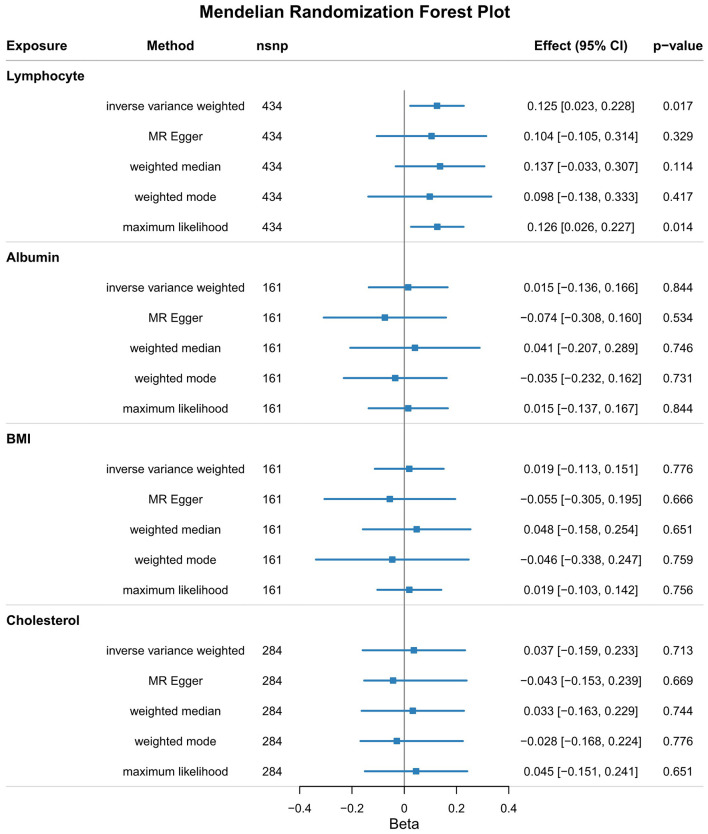
MR showed that genetic pre-disposition to lymphocyte cell count was causally related to the age of predicted TMS30 in HD patients (β = 0.125, 95% CI [0.023, 0.228], *p* = 0.017).

Genetic pre-disposition to lymphocyte cell count was causally related to the age of predicted TMS30 in HD patients (β = 0.125, 95% CI [0.023, 0.228], *p* = 0.017; Figure 2). The alternative MR approaches yielded similar results (maximum likelihood β = 0.126, 95% CI [0.026, 0.227], *p* = 0.014). However, no significant association was found between albumin, BMI, cholesterol, and clinical characteristics of HD (Figure 2). In all analyses, there was no heterogeneity (*I*^2^ < 6% and *p* > 0.05, respectively; Supplementary Table 4) and directional pleiotropy (*p* for MR-Egger intercept >0.2; Supplementary Table 4).

## Discussion

This study is the first to investigate the prevalence of malnutrition, associated factors, and prognostic significance in Chinese HD patients using three validated composite indices—GNRI, PNI, and CONUT. We found that malnutrition is prevalent in HD patients, with the GNRI-based malnutrition strongly correlating with functional decline, cognitive impairment, and advanced disease stage. However, no independent prognostic value of malnutrition was observed for survival. MR revealed that higher lymphocyte counts were directionally associated with delayed motor progression, suggesting potential immunonutritional pathways that may warrant further mechanistic and interventional studies.

Previous studies have shown that malnutrition is more common in patients with PD and MSA than in HCs (17, 21), aligning with our observations in HD. In our HD cohort, the prevalence of malnutrition varied by assessment tool: 34.51% were classified as malnourished using the CONUT vs. 7.96% using the GNRI. This discrepancy is consistent with findings in PD and MSA, where GNRI similarly yields lower malnutrition rates than CONUT (17, 19, 21). The difference likely reflects HD-specific pathophysiology. GNRI incorporates body weight, potentially better reflecting hypermetabolic states driven by chorea-related energy expenditure (9). In HD, negative energy balance and mitochondrial bioenergetic impairment are well-described (34), and a high baseline BMI was associated with a significantly slower rate of functional, motor, and cognitive deterioration, independent of CAG length (10). CONUT relies on lymphocyte count and cholesterol levels, which may be confounded by HD-associated neuroinflammation (24) and peripheral metabolic dysfunction (37, 38), potentially inflating malnutrition estimates. Compared to the CONUT, the GNRI only includes the albumin parameter but lacks the lymphocyte count and total cholesterol level parameters, which may explain the lower prevalence estimated by the GNRI. GNRI may therefore align more closely with the metabolic-functional axis that underpins HD disability trajectories than indices emphasizing circulating lipids or leukocytes alone. The role of body weight in HD also warrants consideration, as its trajectory remains unclear: weight loss may not be inevitable (11), may maintain stable body weight throughout the progression of the disease, or a high BMI at the initial stage of the disease may compensate for subsequent weight loss (19). Additionally, BMI differences between malnutrition classifications by both tools were non-significant in this study, possibly attributable to the cohort's short disease duration. Although the proportion of malnutrition assessed by different assessment tools varied widely, the prevalence of malnutrition in HD remained high overall. Therefore, there is a crucial need to identify reliable screening tools for evaluating malnutrition risk in this population.

Patients classified as malnourished (according to GNRI) exhibited more severe movement disorders, reduced functional capacity, poorer cognitive performance, and advanced disease stages. Excessive movements result in an enhanced catabolic state, meaning that normal or even high-calorie intakes are insufficient to sustain weight (9). Cognitive impairment and motor dysfunction can result in dysphagia and loss of independent feeding (39, 40). Malnutrition in HD is likely multifactorial, including disturbances in energy metabolism, malabsorption, disruption of the microbiome, increased energy requirement because of chorea, as well as lack of nutrition due to dysphagia and psychiatric symptoms (39, 41). Our data suggest that in HD, the component capturing weight relative to ideal—unique to GNRI—adds discriminative information that more accurately reflects daily functional capacity and cognition. This finding is consistent with the field's recognition that weight maintenance is clinically meaningful (10, 42).

Interestingly, there was no independent association between malnutrition and survival outcomes in our cohort. This contrasts with previous studies, which have found that malnutrition often predicts mortality in many systemic diseases (22–24, 43–45). The same nutritional indices (GNRI and PNI in particular) have been used recently in multiple heart failure cohorts to demonstrate strong associations with functional status and mortality, underscoring that these tools capture a generalizable low-reserve, catabolic phenotype across chronic systemic diseases. Two recent works by El-Sheikh et al. (46, 47) in elderly heart failure populations showed that GNRI- and PNI-defined malnutrition was common and prognostically meaningful, and that its effects were modified by BMI and inflammatory status. Li et al. (21) showed an association between malnutrition using the CONUT score and mortality in patients with MSA and MSA-P (21). A study indicated that in individuals with PD, higher levels of CONUT score were linked with increased mortality rates, while higher levels of PNI and GNRI are associated with decreased mortality risk (20). The lack of an independent survival association observed in HD may be due to genetic drivers (e.g., CAG repeat length) and limited event numbers. Additionally, the relatively short median follow-up duration (5.74 years) may be insufficient to capture the long-term nutritional effects of a slowly progressive disease. Larger multicenter studies with long-term follow-up are warranted.

Peripheral immune activation is detectable years before clinical onset in HD (46) and remains elevated throughout the disease course (48). Our MR study demonstrated that better preserved adaptive immunity is associated with slower motor progression, corroborating a prior observational study (27). Functional crosstalk between the peripheral immune system (blood cells) and the central nervous system in the background of a compromised blood-brain barrier (49, 50). Expression of mHTT in peripheral T cells and B cells is associated with disease progression (51). Early neuroinflammation is evidenced by elevated cerebrospinal fluid soluble CD27 receptor (50) and intrathecal T helper 17.1 cells in pre-manifest HD (51). T regulatory cells (Tregs) appear to be protective (52). A skewing in the balance between pro-inflammatory and Tregs may potentially favor a pro-inflammatory intrathecal environment in *HTT* gene expansion carriers (53). The exact role of B lymphocytes in HD pathogenesis remains underexplored. Further studies should delineate adaptive immune mechanisms underlying HD progression.

A key strength is using simple, widely accessible nutritional tools for rapid malnutrition screening in HD. The large sample size of GWAS improves measurement accuracy, and strict instrumental variables (IVs) screening dramatically improves the reliability of the results.

This study has several limitations. First, while we employed well-established nutritional assessment tools, these indices may not fully capture the complexity of malnutrition in HD, particularly in terms of specific nutrient deficiencies or metabolic changes. Future studies could incorporate more comprehensive metabolic assessments. Second, the relatively small sample size may limit the generalizability of our findings, particularly in terms of survival analysis, where a larger cohort and extended follow-up period would provide more robust insights into the long-term effects of malnutrition. Third, the study only evaluated baseline nutritional status without follow-up assessments. The study did not explore changes in nutritional status over time and their relationship with follow-up outcomes. While MR is believed to have the advantage of naturally eliminating confounders, some potential and unknown confounders cannot be eliminated, leading to conclusions that must be viewed cautiously. Additionally, data from the GeM-HD Consortium came from individuals of European descent, and the cohort study was conducted in China. Population heterogeneity may impact the external validity of MR results.

## Conclusion

In conclusion, our study highlights the high prevalence of malnutrition in Chinese HD patients and its association with functional decline and cognitive impairment. Although nutritional indices did not independently predict the composite survival endpoint in our cohort, the MR results suggest that specific immunonutritional components (particularly lymphocyte count) may be relevant to motor progression. These findings support routine nutritional screening in HD and motivate further mechanistic and interventional studies to clarify whether targeting immunonutritional pathways can improve clinical outcomes.

## Data Availability

The raw data supporting the conclusions of this article will be made available by the authors, without undue reservation.
