# Supplementary material for: Clinical implications of malnutrition in Huntington's disease progression: evidence from a Chinese cohort and Mendelian randomization
Source: Front Nutr. 2026 Mar 23;13:1718264. doi: 10.3389/fnut.2026.1718264 (PMC13050713; doi:10.3389/fnut.2026.1718264)
Supplement: Supplementary file 1 [file Table_1.docx]

Supplementary Figure 1: The comparison of survival between Huntington's disease (HD) patients with normal-nutrition and malnutrition by Controlling Nutritional Status score (A) or Geriatric Nutritional Risk Index (B).


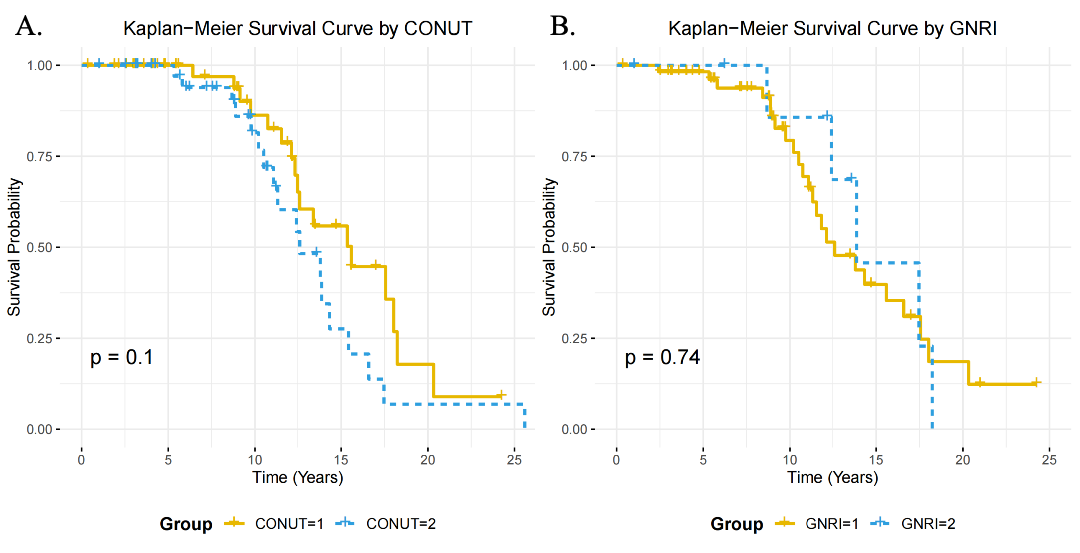


Abbreviations: HD, Huntington's disease; CONUT, Controlling Nutritional Status; GNRI, Geriatric Nutritional Risk Index.

Supplementary Table 1: Detailed information on data sources.

| Phenotype | Ieu/EBI ID | Ref | Consortium | Ancestry | Participants |
| --- | --- | --- | --- | --- | --- |
| ****Exposure**** |  |  |  |  |  |
| Albumin | 30600_irnt | Neale lab | UK biobank | EUR | 432,048 individuals |
| Cholesterol | 30690_irnt | Neale lab | UK biobank | EUR | 470,314 individuals |
| BMI | ieu-b-40 | 30124842 | GIANT- consortium | EUR | 681275 individuals |
| Lymphocyte | ieu-b-32 | 32888494 | Blood Cell Consortium | EUR | 563,946 individuals |
| ****Outcome**** |  |  |  |  |  |
| RAOO | NA | 31398342 | GeM-HD consortium | EUR | 9,064 individuals |
| TFC6 | NA | 31398342 | GeM-HD consortium | EUR | 9,064 individuals |
| TMS30 | NA | 31398342 | GeM-HD consortium | EUR | 9,064 individuals |

Abbreviations: BMI, body mass index; RAOO, residual age of onset; TFC, total functional capacity; TMS, total motor score.

Supplementary Table 2: The associations between malnutrition and HD clinical characteristics by univariate regression models.

| **Clinical Characteristic** | **COUNT score** | | | **GNRI score** | | | | **PNI score** | | |
| --- | --- | --- | --- | --- | --- | --- | --- | --- | --- | --- |
|  | **r** | **95%CI** | **p value** | | **r** | **95%CI** | **p value** | **r** | **95%CI** | **p value** |
| **MMSE** | 0.017 | (-0.224 - 0.256) | 0.889 | | 0.149 | (-0.102 - 0.383) | 0.243 | 0.091 | (-0.144 - 0.316) | 0.449 |
| **HAMD** | -0.099 | (-0.393 - 0.212) | 0.519 | | -0.084 | (-0.372 - 0.218 | 0.587 | 0.023 | (-0.275 - 0.318) | 0.880 |
| **HAMA** | -0.216 | (-0.652 - 0.328) | 0.418 | | -0.210 | (-0.639 - 0.319) | 0.434 | -0.206 | (-0.637 - 0.323) | 0.444 |
| **BDI** | -0.065 | (-0.408 - 0.294) | 0.719 | | -0.013 | (-0.355 - 0.332) | 0.942 | -0.001 | (-0.344 - 0.343) | 0.998 |
| **PBA-s** | -0.017 | (-0.244 - 0.211) | 0.879 | | -0.070 | (-0.310 - 0.179) | 0.584 | 0.036 | (-0.186 - 0.253) | 0.754 |
| **UHDRS-TMS** | 0.044 | (-0.177 - 0.260) | 0.692 | | -0.253 | (-0.469 - -0.007) | ***0.044**** | -0.068 | -0.2763 - 0.146) | 0.531 |
| **UHDRS-TFC** | 0.004 | (-0.215 - 0.223) | 0.969 | | 0.305 | (0.066 - 0.511) | ***0.014**** | 0.087 | (-0.127 - 0.294) | 0.423 |
| **Disease stage** | 0.013 | (-0.207 - 0.232) | 0.906 | | -0.319 | (-0.522 - -0.082) | ***0.009**** | -0.116 | (-0.320 - 0.098) | 0.287 |
| **SDMT** | -0.104 | (-0.344 - 0.148) | 0.405 | | 0.235 | (-0.063 - 0.494) | 0.120 | 0.286 | (0.049 - 0.493) | ***0.019**** |
| **SCN** | -0.018 | (-0.258 - 0.225) | 0.883 | | 0.267 | (-0.018 - 0.513) | 0.066 | 0.179 | (-0.057 - 0.395) | 0.136 |
| **SWR** | 0.062 | (-0.184 - 0.301) | 0.612 | | 0.165 | (-0.129 - 0.431) | 0.269 | 0.209 | (-0.027 - 0.423) | 0.082 |
| **SI** | -0.147 | (-0.375 - 0.098) | 0.225 | | 0.081 | (-0.211 - 0.360) | 0.590 | 0.095 | (-0.142 - 0.321) | 0.433 |
| **cUHDRS** | -0.010 | (-0.262 - 0.243) | 0.937 | | 0.321 | (0.026 - 0.564) | ***0.034**** | 0.231 | (-0.013 - 0.449) | 0.064 |

Abbreviations: CI, Confidence Interval; CONUT, Controlling Nutritional Status; GNRI, Geriatric Nutritional Risk Index; PNI, Prognostic Nutritional Index; UHDRS, Unified Huntington's Disease Rating Scale; TFC, total functional capacity; TMS, total motor score; MMSE, mini–mental state examination; SDMT, symbol digit modality test; SWR, Stroop word reading test; SCN, Stroop color naming test; SI, Stroop interference test; BDI, Beck Depression Inventory; PBA-s, the short version of the Problem-Behavior Assessment; cUHDRS, composite Unified Huntington's Disease Rating Scale; HAMD, Hamilton Depression Scale; HAMA, Hamilton Anxiety Scale.

*P<0.05, **P<0.01, ***P<0.001.

**Supplementary Table 3**: Univariate and multivariate Cox proportional-hazards regression analyses for survival in patients with HD.

| Variables | **Univariate model** | | **Multivariate model (CONUT)** | | **Multivariate model (GNRI)** | |
| --- | --- | --- | --- | --- | --- | --- |
|  | HR (95%CI) | p value | HR (95%CI) | p value | HR (95%CI) | p value |
| Age | 0.861 (0.429- 0.729) | ***0.0003**** | 0.455 (0.323-0.641) | ***<0.0001**** | 0.267(0.156- 0.456) | ***<0.0001**** |
| Sex | 0.946 (0.918- 1.975) | 0.674 | 1.231 (0.496- 3.054) | 0.654 | 0.773(0.295-2.024) | 0.599 |
| Symptoms of onset | 0.975 (0.942-1.008) | 0.141 | 2.112 (1.532- 2.912) | ***<0.0001**** | 3.672 (2.154- 6.258) | ***<0.0001**** |
| Disease duration | 0.813 (0.737-0.897) | ***<0.0001**** | 0.726 (0.560- 0.942) | ***0.016**** | 0.841 (0.673-1.052) | 0.130 |
| CAG repeat | 1.078 (1.023-1.134) | ***0.004**** | 1.095 (0.898-1.334) | 0.371 | 0.990 (0.797-1.231) | 0.930 |
| UHDRS-TMS score | 1.001 (0.98-1.022) | 0.924 | 1.055 (1.018-1.093) | ***0.003**** | 1.040(0.994- 1.089) | 0.093 |
| Malnutrition (CONUT) | 1.752 (0.885-3.467) | 0.108 | 1.362 (0.505-3.675) | 0.542 | 1.463(0.527-4.060) | 0.465 |
| Malnutrition (GNRI) | 0.850 (0.322-2.248) | 0.744 | 0.715 (0.138-3.718) | 0.690 | 0.418(0.081-2.142) | 0.295 |

Abbreviations: CI, Confidence Interval; UHDRS, unified Huntington's disease rating scale; TMS, total motor score; CONUT, Controlling Nutritional Status; GNRI, Geriatric Nutritional Risk Index.

*Significant difference.

**Supplementary Table 4:** Heterogeneity and pleiotropy analysis.

| **Exposure** | **No.SNPs in MR** | **IVW-Q test** | | **MR-Egger** | | |
| --- | --- | --- | --- | --- | --- | --- |
|  |  | **Q-statistic** | **P for heterogeneity** | **Egger intercept** | **P for pleiotropy** | **P for heterogeneity** |
| **Outcome of RAOO** |  |  |  |  |  |  |
| **lymphocyte** | 434 | 464.3666853 | 0.143731311 | -0.002295853 | 0.867763585 | 0.136461304 |
| **albumin** | 161 | 150.1108894 | 0.700959505 | 0.002668829 | 0.894663178 | 0.681369799 |
| **BMI** | 161 | 477.5680432 | 0.14566342 | -0.012189997 | 0.439796357 | 0.142794387 |
| **Cholesterol** | 284 | 372.8451926 | 0.184927361 | -0.004613872 | 0.612845739 | 0.176203584 |
| **Outcome of TFC6** |  |  |  |  |  |  |
| **lymphocyte** | 434 | 420.1598085 | 0.662027669 | 0.000905343 | 0.656094346 | 0.65199005 |
| **albumin** | 161 | 193.5016288 | 0.056509481 | 0.002693885 | 0.426719903 | 0.055221605 |
| **BMI** | 161 | 427.8600679 | 0.723544822 | -0.002993621 | 0.200034963 | 0.73118398 |
| **Cholesterol** | 284 | 205.6384179 | 0.058734912 | 0.001978436 | 0.431290665 | 0.061482907 |
| **Outcome of TMS30** |  |  |  |  |  |  |
| **lymphocyte** | 434 | 454.5987643 | 0.22823878 | 0.00058751 | 0.821364334 | 0.218687434 |
| **albumin** | 161 | 188.0859358 | 0.063836913 | 0.00232288 | 0.494150491 | 0.060561703 |
| **BMI** | 161 | 449.0082353 | 0.451129118 | -0.002071964 | 0.383594311 | 0.447988032 |
| **Cholesterol** | 284 | 428.3097612 | 0.721563904 | -0.001965337 | 0.214508996 | 0.726884113 |

Abbreviations: RAOO, residual age of onset; BMI, body mass index; TFC, total functional capacity; TMS, total motor score; MR, Mendelian randomization; SNP, single nucleotide polymorphism; IVW, Inverse variance weighted.
